# Universal scaling laws for charge-carrier interactions with quantum confinement in lead-halide perovskites

**DOI:** 10.1038/s41467-023-35842-4

**Published:** 2023-01-16

**Authors:** Philippe Tamarat, Elise Prin, Yuliia Berezovska, Anastasiia Moskalenko, Thi Phuc Tan Nguyen, Chenghui Xia, Lei Hou, Jean-Baptiste Trebbia, Marios Zacharias, Laurent Pedesseau, Claudine Katan, Maryna I. Bodnarchuk, Maksym V. Kovalenko, Jacky Even, Brahim Lounis

**Affiliations:** 1 Université de Bordeaux, LP2N, Talence, F-33405 France; 2 Institut d’Optique and CNRS, LP2N, Talence, F-33405 France; 3 Empa-Swiss Federal Laboratories for Materials Science and Technology, CH-8600 Dübendorf, Switzerland; 4 Institute of Inorganic Chemistry, Department of Chemistry and Applied Biosciences, ETH Zürich, CH-8093 Zürich, Switzerland; 5 Univ Rennes, ENSCR, CNRS, ISCR-UMR 6226, Rennes, F-35000 France; 6 Univ Rennes, INSA Rennes, CNRS, Institut FOTON—UMR 6082, F-35000 Rennes, France

**Keywords:** Nanoparticles, Electronic properties and materials, Quantum dots

## Abstract

Lead halide perovskites open great prospects for optoelectronics and a wealth of potential applications in quantum optical and spin-based technologies. Precise knowledge of the fundamental optical and spin properties of charge-carrier complexes at the origin of their luminescence is crucial in view of the development of these applications. On nearly bulk Cesium-Lead-Bromide single perovskite nanocrystals, which are the test bench materials for next-generation devices as well as theoretical modeling, we perform low temperature magneto-optical spectroscopy to reveal their entire band-edge exciton fine structure and charge-complex binding energies. We demonstrate that the ground exciton state is dark and lays several millielectronvolts below the lowest bright exciton sublevels, which settles the debate on the bright-dark exciton level ordering in these materials. More importantly, combining these results with spectroscopic measurements on various perovskite nanocrystal compounds, we show evidence for universal scaling laws relating the exciton fine structure splitting, the trion and biexciton binding energies to the band-edge exciton energy in lead-halide perovskite nanostructures, regardless of their chemical composition. These scaling laws solely based on quantum confinement effects and dimensionless energies offer a general predictive picture for the interaction energies within charge-carrier complexes photo-generated in these emerging semiconductor nanostructures.

## Abstract (web summary)

Knowledge of fundamental properties of lead halide perovskites is crucial for their technological development. Here, authors perform magneto-optical spectroscopy, develop universal scaling laws and offer a predictive picture for interaction energies within photo-generated charge-carrier complexes.

## Introduction

Lead halide perovskites have recently emerged as one of the most promising materials for a broad range of applications ranging from photovoltaics to optoelectronics^1–4^ and potential applications as quantum light sources^5–10^. While intense experimental and theoretical efforts have been made to explore their unique optical and electronic properties, the physics of band-edge charge complexes (e.g. excitons, trions, biexcitons), whose recombination is at the origin of the luminescence, remains unclear.

For instance, the ordering of bright and dark sublevels in the exciton fine structure is a subject of ongoing debate, though essential for the development of quantum light sources^11–13^ and for spin-based technologies^14–16^. The band-edge exciton of perovskites is expected to split by electron-hole exchange interaction into a dark ground singlet state (with zero angular momentum) and a bright triplet (with angular momentum unity)^17–20^. However, since cesium lead halide perovskite nanocrystals (NCs) exhibit strong photoluminescence (PL) with triplet-line emission at cryogenic temperatures^19,21–24^, it has been proposed that a Rashba effect reverses the fine structure level ordering and places the dark singlet above the bright triplet^24–27^, making perovskites an exception among all bulk semiconductors and all existing semiconductor heterostructures. Studies on Mn-doped and undoped ensembles of CsPbCl_3_ NCs^28^ have suggested that the dark state is positioned below the bright triplet and that a slow bright–dark state relaxation at cryogenic temperatures gives rise to almost exclusively bright-state emission. Then, low-temperature single-NC magneto-optical spectroscopy^29^ has provided direct signatures of the dark singlet exciton emission, showing that the singlet is positioned few meV below the bright triplet in hybrid organic–inorganic formamidinium lead bromide (FAPbBr_3_)^30^ and inorganic cesium lead iodide (CsPbI_3_)^9^ NCs, in agreement with theoretical estimations of confined exchange interaction.

Similarly, no general picture is available for many-body Coulomb interactions in perovskites, besides investigations of the size dependence of the biexciton and trion binding energies in single FAPbBr_3_ NCs^31^. The biexciton binding energy has important consequences in the development of perovskite-based light-emitting technologies, since a large shift of the biexciton recombination lines with respect to those of the band-edge exciton would reduce the emission color purity under high-flux excitation. Its sign also fixes the transition that may sustain population inversion and NC gain for lasing applications^32^. For CsPbBr_3_ perovskites, contradictory results have been reported on the amplitude and sign of the biexciton energy shift in the range −100 meV to 100 meV, using time- and spectrally- resolved PL^33–35^, transient absorption^36–39^ or nonlinear^40^ spectroscopic methods on NC ensembles. Heralded single-NC spectroscopy on CsPbBr_3_ and CsPbI_3_ perovskites lead to estimations of the biexciton binding energy at room-temperature with inherently low spectral precision and even uncertainties on the sign of the interaction^41^.

Here, we use low-temperature single-NC magneto-optical spectroscopy to reveal the entire band-edge exciton fine structure and charge-complex binding energies of nearly bulk CsPbBr_3_ perovskite NCs, the reference materials for emerging applications and theoretical modeling. We demonstrate that the dark singlet exciton lays at least 3.6 meV below the bright triplet, at variance with the models based on the Rashba effect^24–26^. This settles the debate on the bright-dark exciton level ordering in halide perovskite nanostructures. Moreover, the spectral fingerprint of biexciton recombination is also evidenced in these materials and provides precise measurements of the biexciton binding energy. Importantly, we show evidence for universal scaling laws that relate the exciton fine structure splitting, the trion and biexciton binding energies to the band-edge exciton energy in lead-halide perovskite nanostructures. For that purpose, we combine the present results on CsPbBr_3_ single NCs with spectroscopic data on various perovskite NCs previously investigated^9,21,22,24,30,31,42^. Additional measurements on single CsPb(Cl_x_Br_1-x_)_3_ NCs further extend the validity of these dependences over a broader emission spectral range. These scaling laws solely based on quantum confinement effects and dimensionless energies offer a general predictive picture for the interaction energies within charge complexes photo-generated in lead halide perovskites, regardless of their chemical composition.

## Results

### Tailoring nearly-bulk CsPbBr3 NCs

**Fig. 1:**
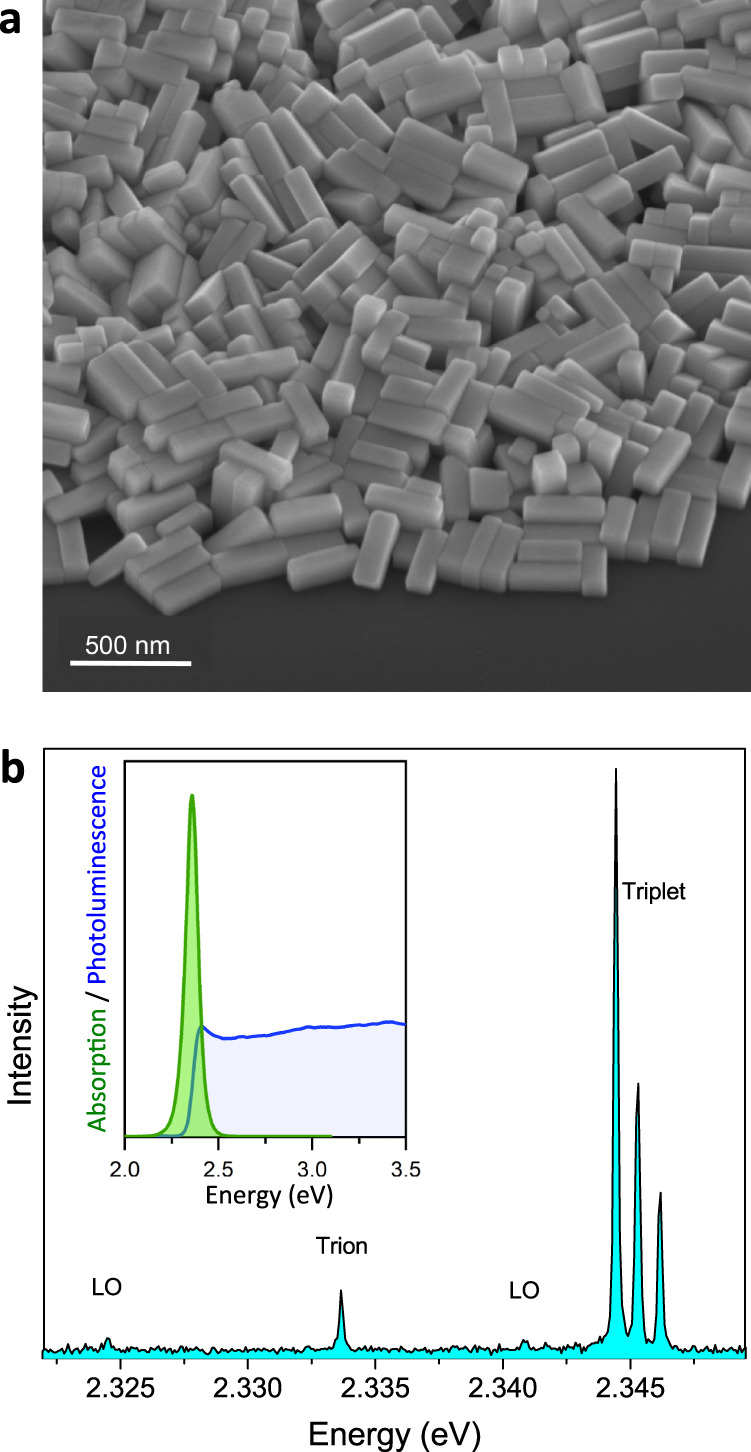
Characterization of the large OGB-capped CsPbBr_3_ NCs. **a** 45°-Scanning electron microscopy image of the OGB-capped CsPbBr_3_ NCs. Their room-temperature absorption and emission spectrum is shown in the inset of **b**. **b** Representative PL spectrum of a single NC at 3.5 K under laser excitation with wavelength 488 nm and intensity 50 W cm^−2^ (integration time 10 s). It displays the bright exciton triplet ZPLs, the trion recombination line as well as longitudinal optical (LO) phonon replicas.

Magnetic brightening of the dark exciton state is challenging in CsPbBr_3_ perovskite NCs. Indeed, the PL intensity of the dark state is inversely proportional to the square of the bright-dark energy splitting, while the bulk electron-hole exchange interaction in this material is particularly strong among lead-halide perovskites^9^. Moreover, confinement effects in NCs enhance the exciton fine structure splittings, which makes magnetic brightening of the dark singlet even more difficult. The CsPbBr_3_ NCs specifically prepared for this study are cuboids with sides that largely exceed the bulk exciton Bohr radius ${a}_{{{{{{\rm{B}}}}}}}$ ~3.1 nm in order to minimize quantum confinement effects on the dark-bright exciton splittings. These large NCs are synthesized under ambient conditions with the ligand-assisted reprecipitation approach, using a novel ligand molecule, oleylguanidinium bromide (OGB), to obtain dispersible and large CsPbBr_3_ perovskite NCs (See Methods, Supplementary Figs. 1 and 2 for details of the NC synthesis and characterization). The 3D size and shape of these OGB-capped CsPbBr_3_ NCs with orthorhombic crystal phase are characterized using 45°-tilted scanning electron microscopy (Fig. 1a). With average base lengths 195 ± 18 nm, 41 ± 8.5 nm and a thickness of ~40 nm, these NCs are nearly bulk-like particles with reduced quantum confinement. The ensemble absorption spectrum presents a threshold at ~520 nm (inset Fig. 1b), which roughly corresponds to the room-temperature bulk electronic bandgap. Under light excitation at 405 nm, these NCs exhibit a bright green PL centered at 2.36 eV, with a high color purity (full width at half-maximum, FWHM, of 77 meV) and a PL quantum yield of 32 % (see inset Fig. 1b).

### Band-edge exciton fine structure

At low temperature ($3.5\,\pm 0.3$ K) and under continuous-wave laser excitation with a wavelength of 488 nm (2.54 eV), the PL spectra of single NCs are characterized by a triplet of resolution-limited zero-phonon lines (ZPLs) corresponding to the recombination of the bright triplet exciton, followed by optical phonon replica, as exemplified on Fig. 1b. The photon energy of these recombination lines is distributed in the range 2.3-2.35 eV, i.e. across the low-temperature electronic bandgap$\,{E}_{{{{{{\rm{gap}}}}}}}$ = 2.342 eV of bulk CsPbBr_3_^43^. The spread of emission energies among the nearly-bulk CsPbBr_3_ NCs may be due to stochastic deviations of cation positions or torsions of the halogen octahedra, which translate into a complex potential landscape felt by the exciton. This is a consequence of the very strong lattice anharmonicity or intrinsic polymorphous nature of the perovskite structure^44–46^. In order to get an overview on the general consequences of these fluctuations, a theoretical framework is proposed in Supplementary Note 1 to show the smearing of the electronic density of states, which leads to the broadening of the energy distribution of the emission lines.

All triplets observed in this study are non-degenerate, which is consistent with triplet splittings mainly set by the NC anisotropic crystal phase for such large crystals^21,47,48^. Indeed, the triplet splittings between extreme lines range from 0.5 to 2.3 meV (Supplementary Fig. 3), with an average of ~1.2 meV that matches the theoretical estimations for nearly-bulk CsPbBr_3_ NCs with an orthorhombic crystal structure^48^. One can also notice on Supplementary Fig. 3 that the triplet splittings have a slight correlation with the NC emission energies, which points to residual effects of exciton quantum confinement. The observation of triplet emission in perovskite NCs indicates that acoustic-phonon-assisted relaxation between triplet sublevels is much longer than the exciton recombination lifetime^49^. This is due to the strong lattice anharmonicity in these soft materials, where the acoustic vibrational density of states is that of a glassy state.^50^

**Fig. 2:**
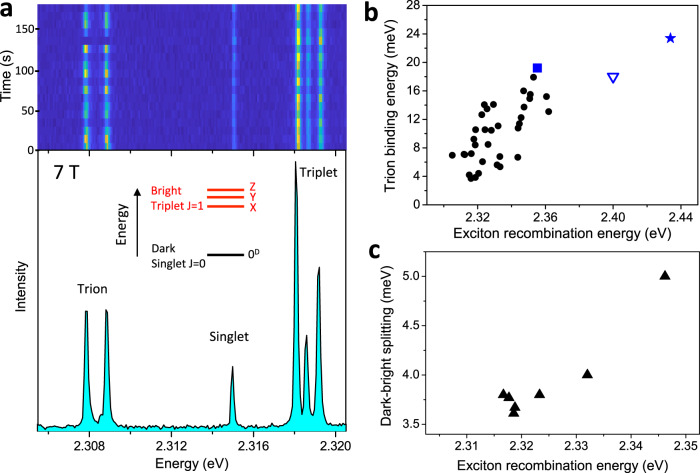
Band-edge exciton fine structure and trion binding energy in CsPbBr_3_ NCs. **a** The upper panel shows the spectral trail of a single NC at 7 T, showing switches between the entire exciton fine structure manifold (triplet + singlet) and the trion Zeeman doublet. The singlet and triplet lines clearly undergo identical variations of their PL intensities. The intensity variations of the doublet and the exciton manifold are anti-correlated, showing evidence that they belong to different charge complexes of the same NC. The lower panel displays the spectrum integrated over the spectral trail. The inset indicates the band-edge exciton fine-structure sublevels, where X, Y, Z arbitrarily refer to linear and orthogonal emission dipoles associated with the NC axes. **b** Distribution of trion binding energies in CsPbBr_3_ single NCs as a function of the exciton recombination energy taken at the central triplet ZPL. The black dots correspond to nearly-bulk NCs, the blue triangle to 9 nm sized NCs^21^. The blue star and the blue square respectively correspond to 5.5 nm sized and 14.5 nm sized NCs (Supplementary Fig. 5). **c** Dark-bright exciton splittings of nearly-bulk CsPbBr_3_ NCs as a function of the exciton recombination energy.

For most of the NCs, the emission switches over time between the neutral exciton triplet and trion emission characterized by a single, red-shifted ZPL that invariably splits into two Zeeman components under the application of an external magnetic field, as shown in Fig. 2a. The $g$-factor associated with the trion doublet is narrowly distributed around 2.4 (Supplementary Fig. 4). This value is in broad agreement with the sum of electron and hole $g$-factors measured on CsPbBr_3_ crystals with time-resolved Kerr rotation^51^. It is also very similar to the trion $g$-factors measured for ~10 nm sized CsPbBr_3_ NCs^21^, FAPbBr_3_ NCs^30^ and CsPbI_3_ NCs^9^. Overall, these results point to a nearly isotropic character of the magnetic response of charged perovskite NCs, regardless of their size and chemical composition. The red shift of the trion line from the central triplet line provides a measure of the trion binding energy and spans from 3 to 18 meV among the studied NCs (Fig. 2b). A correlation emerges between the trion binding energy and the exciton recombination energy (Fig. 2a). This correlation becomes obvious when adding to this set of data the trion binding energy measured on smaller CsPbBr_3_ NCs having sizes 14.5 nm, 9 nm and 5.5 nm (Fig. 2b). Their corresponding low-temperature emission energies are respectively 2.355 eV, ~2.40 eV^21^ and 2.434 eV, as a signature of increasing charge-carrier confinement.

### Bright triplet—dark singlet level ordering

The application of a magnetic field also reveals the entire band-edge exciton fine structure of the nearly-bulk CsPbBr_3_ NCs, as shown in Fig. 2a. While the triplet further splits under increasing fields, a red-shifted line indeed develops in the PL spectrum as a hallmark of magnetic brightening of the ground dark singlet exciton state (See also Supplementary Fig. 6). The ZPL of the dark state at fields limited to 7 T is often very weak and its observation requires PL spectra with a remarkable quality, which requires excellent photostability and long acquisitions times. The dark singlet lays 3.6 meV to 5 meV below the middle bright triplet sublevel in these CsPbBr_3_ NCs (Fig. 2c). These results invalidate recent models of exciton fine structure, which predict for weakly confined CsPbBr_3_ NCs a bright-dark exciton level inversion caused by the Rashba effect^24–26^. The strong triplet luminescence of lead halide bulk-like perovskites NCs at liquid helium temperatures is actually a signature of reduced phonon-assisted relaxation to the dark ground exciton, even if the dark-bright splittings coincides with the LO-phonon energies. Triplet-to-singlet relaxation with a LO-phonon is indeed inhibited due to the fact that phonons do not carry angular momentum^52,53^. The ground singlet exciton can nevertheless be populated by relaxation from high-energy continuum states excited at 2.54 eV, with important consequences on the statistics of photons emitted by individual bulk-like NCs. As a matter of fact, strong photon bunching is evidenced in the low-temperature PL intensity autocorrelation function of these NCs (Supplementary Fig. 7), while a flat correlation is expected for bulk-like particles with Poissonian photon statistics. Magnetic coupling of dark and bright states leads to shortening of the long component of the PL decay and to weakening of the photon bunching, as shown in Supplementary Fig. 7 and modeled in Supplementary Fig. 8. These observations indicate that the population of the long-lived ground exciton state favors the formation of biexcitons in perovskites^9^.

### Biexciton binding energy

**Fig. 3:**
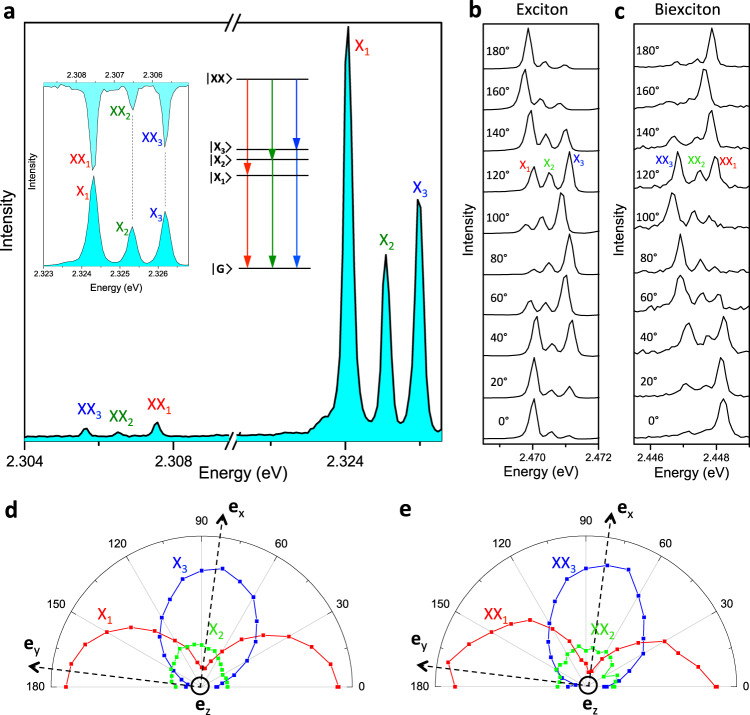
Biexciton-to-exciton transitions and biexciton binding energy in perovskite NCs. **a** Low-temperature PL spectrum of a single CsPbBr_3_ NC at high excitation intensity (50 kW cm^−2^) and 7 T. The lines named X_1_, X_2_, X_3_ are assigned to the exciton triplet recombination ZPLs. The red-shifted lines named XX_1_, XX_2_, XX_3_ are assigned to the biexciton-to-exciton transitions and perfectly map onto the exciton triplet ZPLs under mirror image (left inset), allowing a straightforward correspondence of the transition lines (right inset). The spectral trail of this NC over time is shown in Supplementary Fig. 9. **b**, **c** Polarized PL spectra zoomed on the exciton triplet ZPLs (**b**) and the biexciton recombination ZPLs (**c**) for a single CsPb(Cl_x_Br_1-x_)_3_ single NC with x~0.3 and size ~30 nm, for various analyzer angles ranging from 0° to 180°. The whole series of spectra is displayed in Supplementary Fig. 12. The evolutions of the exciton and biexciton ZPL-intensities as a function of the polarizer angle are displayed in the polar plots **d** and **e**, respectively. These polar plots are remarkably similar, demonstrating that X_1_ and XX_1_, X_2_ and XX_2_, X_3_ and XX_3_ have identical transition dipole orientations nearly aligned along the orthogonal unit vectors ${{{{{{\bf{e}}}}}}}_{{{{{{\rm{Y}}}}}}}$, ${{{{{{\bf{e}}}}}}}_{{{{{{\rm{Z}}}}}}}$, ${{{{{{\bf{e}}}}}}}_{{{{{{\rm{X}}}}}}}$, respectively.

**Fig. 4:**
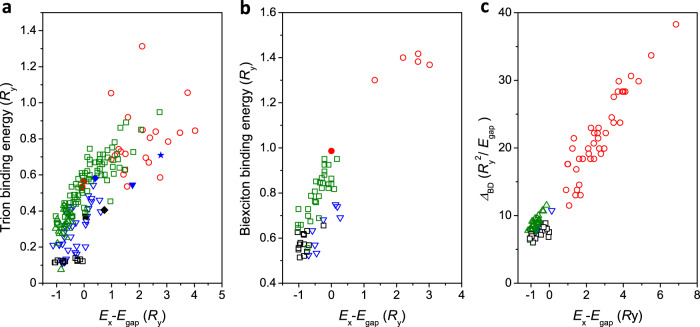
Scaling laws of charge-complex energies in lead-halide perovskite single NCs. Evolutions of the single-NC trion binding energies in ${R}_{{{{{{\rm{y}}}}}}}$ units (**a**), biexciton binding energies in ${R}_{{{{{{\rm{y}}}}}}}$ units (**b**) and dark-bright exciton energy splittings in ${{R}_{{{{{{\rm{y}}}}}}}}^{2}/{E}_{{{{{{\rm{gap}}}}}}}$ units (**c**) as a function of the exciton recombination energy expressed in ${R}_{{{{{{\rm{y}}}}}}}$ units after bandgap subtraction. The splittings and binding energies are measured with respect to the central line of the exciton triplet. Open blue triangles: CsPbBr_3_ NCs; Open black squares: CsPb(Cl_x_Br_1-x_)_3_ NCs; Green triangles: FAPbBr_3_ NCs^30^; Red circles: CsPbI_3_ NCs^9^. Green squares: FAPbBr_3_ NCs (data extracted from ref. ^31^). Plain blue triangle in (**a**): average data for 9 nm sized CsPbBr_3_ NCs^21^. Blue star in (**a**): CsPbBr_3_ NC with 5.5 nm size. Blue diamond in (**a**): CsPbBr_3_ NC with 14.5 nm size. Plain black square in (**a**): CsPbClBr_2_ NCs from ref. ^24^. Black diamond in (**a**): CsPb(ClBr)_3_ NCs from ref. ^42^. Red disks in (**a**) and (**b**): CsPbI_3_ NCs^22^. For these perovskites, the low-temperature bandgaps and bulk exciton binding energies taken for the normalization are deduced from refs. ^43,62,63^ (See Supplementary Table 1): ${E}_{{{{{{\rm{gap}}}}}}}$ = 2.342 eV and ${R}_{{{{{{\rm{y}}}}}}}$ = 33 meV for CsPbBr_3_, ${E}_{{{{{{\rm{gap}}}}}}}$ = 1.723 eV and ${R}_{{{{{{\rm{y}}}}}}}$ = 15 meV for CsPbI_3_, ${E}_{{{{{{\rm{gap}}}}}}}$ = 2.233 eV and ${R}_{{{{{{\rm{y}}}}}}}$ = 24 meV for FAPbBr_3_, ${E}_{{{{{{\rm{gap}}}}}}}$ = 2.513 eV and ${R}_{{{{{{\rm{y}}}}}}}$ = 42 meV for CsPb(Cl_x_Br_1-x_)_3_ with x = 0.24. This value of x was adjusted using empirical interpolations of the bandgaps of bulk CsPbCl_3_ and CsPbBr_3_ materials composing the alloy. It matches reasonably well the estimation x~0.3 after the growth procedure (see Methods). The trion binding energies in **a** experience a larger spread than the biexciton ones in **b**, as a result of spectral jumps accompanying the neutral-to-charged switches of the NCs, as illustrated in Supplementary Fig. 9.

Our spectroscopic investigations of single perovskite NCs also reveal the spectral fingerprint of the biexciton-to-exciton transitions. Indeed, raising the excitation intensity leads to the onset of a spectral triplet (XX) that is red-shifted with respect to the exciton (X) bright triplet (Fig. 3a), and whose temporal variations in PL intensity are correlated with those of the X triplet (Supplementary Fig. 9). Moreover, the ratio of the integrated intensities of the XX and X triplets is proportional to the intensity of the X triplet, as a distinctive signature of XX biexciton emission^22,54^ (Supplementary Fig. 10). Additionally, the XX triplet components XX_i_ perfectly match the X triplet components X_i_ (i = 1,2,3) under mirror symmetry and are attributed to the three transitions that link the ground zero-angular-momentum biexciton state to the bright exciton triplet sublevels (Fig. 3a). Further evidence of the correspondence between the XX_i_ and X_i_ lines comes from their polarizations, as shown on Fig. 4b–e for another perovskite NC. For this NC, the transition dipoles associated to X_1_ and X_3_ (as well as XX_1_ and XX_3_), have nearly linear and orthogonal polarizations in the focal plane, while X_2_ (as well as XX_2_) with weak intensity and polarization character is assigned to a transition dipole oriented along the optical axis. This demonstrates that XX_i_ and X_i_ transitions involving the same bright state have identical polarizations, as expected from symmetry considerations (Supplementary Note 2).

Importantly, the red shift of the XX triplet with respect to the X triplet indicates that the biexciton interaction is attractive. In the large CsPbBr_3_ particles, the biexciton binding energy ranges from 17 to 25 meV with a trend of correlation with the corresponding exciton recombination energy (Supplementary Fig. 11), as in the case of trion emission. These measurements provide the bulk CsPbBr_3_ biexciton binding energy ~17 meV, a key parameter for the development of refined models of many-body Coulomb interactions in perovskites^55^. This value is in good agreement with that extrapolated from two-dimensional electronic spectroscopic measurements performed on small CsPbBr_3_ NC ensembles^40^.

In order to strengthen the generality of these findings, we have also conducted magneto-optical spectroscopic investigations of mixed-halide CsPb(Cl_x_Br_1-x_)_3_ single NCs with an average size ~30 nm (See Methods). These perovskites follow the same behaviors as the other compounds in terms of exciton sublevel ordering and signs of the interactions within trion and biexciton charge complexes. Their exciton dark-bright splittings are found in the range 4-6 meV, while their trion and biexciton binding energies are respectively of the order of 5 meV and 24 meV (Supplementary Fig. 13).

## Discussion

We now aim at giving a general and predictive picture for the exciton dark-bright energy splittings as well as the trion and biexciton binding energies in lead halide perovskite NCs from various compositions, on the basis of single-NC spectroscopic data gathered in this study and from previous reports^9,21,22,24,30,31,42^. As compared to II-VI and III-V semiconductor, perovskites exhibit a simpler band structure (a single valence band, and a single conduction band due to the giant spin-orbit coupling). Moreover, for each perovskite compound, the electron and the hole have nearly identical and effective masses ${m}^{*}$^18,24^ and confinement potentials. The Hamiltonian ${{{{{\mathcal{H}}}}}}$ describing the kinetic and Coulomb energies of the charge carriers in a NC, where the confinement potential with infinitely high barriers is taken to zero, can be written

1 $${{{{{\mathcal{H}}}}}} 	=\mathop{\sum}\limits_{{{{{{\rm{A}}}}}}}\frac{-{{{\hslash }}}^{2}}{2{m}^{*}{a}_{{{{{{\rm{B}}}}}}}^{2}}\frac{1}{{\left({L}_{{{{{{\rm{eff}}}}}}}/{a}_{{{{{{\rm{B}}}}}}}\right)}^{2}}{\hat{{{{{{\boldsymbol{\nabla }}}}}}}}_{{{{{{\rm{A}}}}}}}^{2}\,+\,\mathop{\sum}\limits_{{{{{{\rm{A}}}}}} < {{{{{\rm{B}}}}}}}\,\frac{{e}^{2}}{4{{{{{\rm{\pi }}}}}}{{{{{{\rm{\varepsilon }}}}}}}_{0}\varepsilon {a}_{{{{{{\rm{B}}}}}}}}\frac{{c}_{{{{{{\rm{A}}}}}}}{c}_{{{{{{\rm{B}}}}}}}}{\left({L}_{{{{{{\rm{eff}}}}}}}/{a}_{{{{{{\rm{B}}}}}}}\right)}\,\frac{1}{\left|{\hat{{{{{{\bf{r}}}}}}}}_{{{{{{\rm{A}}}}}}}-{\hat{{{{{{\bf{r}}}}}}}}_{{{{{{\rm{B}}}}}}}\right|} \\ 	={R}_{{{{{{\rm{y}}}}}}}\,\left(\mathop{\sum}\limits_{{{{{{\rm{A}}}}}}}\frac{-1}{{2\left({L}_{{{{{{\rm{eff}}}}}}}/{a}_{{{{{{\rm{B}}}}}}}\right)}^{2}}{\hat{{{{{{\boldsymbol{\nabla }}}}}}}}_{{{{{{\rm{A}}}}}}}^{2}+\,\frac{2}{\left({L}_{{{{{{\rm{eff}}}}}}}/{a}_{{{{{{\rm{B}}}}}}}\right)}\,\mathop{\sum}\limits_{{{{{{\rm{A}}}}}} < {{{{{\rm{B}}}}}}}{c}_{{{{{{\rm{A}}}}}}}{c}_{{{{{{\rm{B}}}}}}}\frac{1}{\left|{\hat{{{{{{\bf{r}}}}}}}}_{{{{{{\rm{A}}}}}}}-{\hat{{{{{{\bf{r}}}}}}}}_{{{{{{\rm{B}}}}}}}\right|}\right)$$

where ${L}_{{{{{{\rm{eff}}}}}}}$ is the effective NC confinement size, ${\hat{{{{{{\boldsymbol{\nabla }}}}}}}}_{{{{{{\rm{i}}}}}}}$ and ${\hat{{{{{{\bf{r}}}}}}}}_{{{{{{\rm{i}}}}}}}$ the dimensionless (normalized to ${L}_{{{{{{\rm{eff}}}}}}}$) Nabla-operators and coordinates of the charge carriers i (i = A, B…), ${c}_{{{{{{\rm{i}}}}}}}$ the signs of their charges having absolute value $e$, and ${R}_{{{{{{\rm{y}}}}}}}={\hslash }^{2}/\left({m}^{*}{a}_{{{{{{\rm{B}}}}}}}^{2}\right)$ the bulk exciton Rydberg energy, $\varepsilon$ being the perovskite dielectric constant. Such normalization in Rydberg’s units makes interaction energies independent of the material and shows that trion and biexciton binding energies are essentially set by the quantum confinement regime, i.e. the dimensionless ratio ${L}_{{{{{{\rm{eff}}}}}}}/{a}_{{{{{{\rm{B}}}}}}}$. This simple statement explains the universal laws relating in Rydberg’s units the experimental trion and biexciton binding energies to the exciton recombination energy ${E}_{{{{{{\rm{X}}}}}}}$, once the bandgap of the material is subtracted (Fig. 4a, b). Indeed, using these scales, the experimental points strikingly spread along a trend line from the bulk limit (abscissa approaching −1) to the strong confinement regime (abscissa of several units), regardless of the perovskite compositions.

The dark-bright exciton energy splitting ${\varDelta }_{{{{{{\rm{BD}}}}}}}$ measured on single NCs of CsPbBr_3_, CsPb(Cl_x_Br_1-x_)_3_, CsPbI_3_^9^ and FAPbBr_3_^30^ also displays a universal scaling law as a function of the exciton quantum confinement. This splitting mainly set by the long-range contribution of the exchange interaction is directly related to the energy difference between longitudinal and transverse excitons in bulk semiconductors, which is proportional to ${{R}_{{{{{{\rm{y}}}}}}}}^{2}/{E}_{{{{{{\rm{gap}}}}}}}$ (Supplementary Note 1). Adding the short-range contribution^20^, we show in the Supplementary Table 2 that the total exchange interaction expected for bulk perovskites with various compositions converge to nearly equal values, once normalized with ${{R}_{{{{{{\rm{y}}}}}}}}^{2}/{E}_{{{{{{\rm{gap}}}}}}}$. Moreover, the short-range and long-range contributions of the exchange interaction are predicted to have the same NC size-dependence in the strongly confined regime^56^. We have thus plotted the normalized splittings ${\varDelta }_{{{{{{\rm{BD}}}}}}}/\left(\frac{{{R}_{{{{{{\rm{y}}}}}}}}^{2}}{{E}_{{{{{{\rm{gap}}}}}}}}\right)$ of all these NCs as a function of the corresponding exciton recombination energies in Rydberg’s units, after bandgap subtraction (Fig. 4c). This figure shows evidence that the data points line up on a same curve, regardless of the cation and halide contained in the perovskite.

Overall, the dimensionless experimental dark-bright exciton splittings as well as trion and biexciton binding energies are evidenced to display a universal behavior mainly set by the quantum confinement of the charge carriers in perovskite nanostructures, without the need for prior characterization of the NC size or composition. Beyond settling the debate on the bright and dark exciton sublevel ordering and its main origin in lead halide perovskites, the laws unraveled by single-NC spectroscopy offer a general predictive picture for the interaction energies within charge complexes photo-generated in these materials. They also pave the way to the development of accurate models of charge complex interactions in lead halide perovskites, which share common physics and form a unique class of semiconductors.

## Methods

### Synthesis of N-(octadec-9-en-1-yl)guanidinium hydrobromide (oleylguanidinium bromide, OGB) ligands

Oleylamine (1 eq) was added to S-ethylisothiourea hydrobromide (1.2 eq) suspended in tetrahydrofurane (THF). The resulting mixture was stirred at room temperature overnight. Then THF was evaporated under reduced pressure and the resulting residue was recrystallized with diethylether (Et_2_O). The obtained precipitate was washed several times with Et_2_O and dried under reduced pressure overnight at 50 °C.

N-(octadec-9-en-1-yl)guanidinium hydrobromide (3, OGB). Yield 60%; white solid. ^1^H NMR (400 MHz, DMSO) δ, ppm ( *J*, Hz): 7.48(t, J = 5.6 Hz, 1H), 7.20 (s, 2H), 6.86 (s, 1H), 5.34 (dt, *J* = 14.7, 4.3 Hz, 1H), 3.09 (q, *J* = 6.6 Hz, 1H), 1.98 (q, *J* = 6.1 Hz, 1H), 1.93 (t, *J* = 5.8 Hz, 1H), 1.44 (q, *J* = 7.0 Hz, 2H), 1.25 (d, *J* = 9.5 Hz, 18H), 0.86 (s, 1H), 0.86 (d, *J* = 13.5 Hz, 1H). ^13^C NMR (101 MHz, DMSO) δ, ppm:157.19; 130.54; 130.51; 130.11; 130.07; 41.20; 32.41; 31.75; 29.60; 29.56; 29.52; 29.47; 29.44; 29.32; 29.29; 29.18; 29.16; 29.08; 29.06; 28.94; 28.91; 27.08; 27.04; 26.52; 22.56; 14.41.

The structural formula of OGB is displayed in Supplementary Fig. 1.

### Ligand-assisted-reprecipitation synthesis of OGB-capped CsPbBr_3_ nanocrystals

The hybrid precursor solution was prepared by mixing solutions of 90 µL of PbBr_2_ (0.67 M in DMF), 465 µL of CsBr (0.043 M in DMF-DMSO in ratio 1:1) and 300 µL of OGB (1 M in DMF), where DMF denotes dimethyl formamide and DMSO dimethyl sulfoxide. 75 µL of the hybrid precursor solution was injected into a 4 mL vial containing 2.5 mL of vigorously stirred mesitylene to initiate fast nucleation of the CsPbBr_3_ NRs. The green solution with bright green luminance could be observed in 15-20 s. For purification of the CsPbBr_3_ NCs, 0.25 mL of ethyl acetate was added to 0.5 mL of the crude solution, followed by centrifugation for 4 min at 4250 $\times$ g. The supernatant was discarded and the precipitate was dispersed in 0.4 mL of toluene.

The synthesis and characterization of the OGB-capped CsPbBr_3_ NCs are depicted on Supplementary Fig. 2.

The solution of CsPbBr_3_ NCs was then diluted 50 times into a solution of polystyrene (1 wt%) in toluene, and 25 µL of the resulting solution was spin-coated on a clean 1 cm x 1 cm sapphire substrate with a rotation speed of 2000 rpm for 60 s, for low-temperature magneto-optical spectroscopic measurements.

### Synthesis of CsPb(Cl_x_Br_1-x_)_3_ NCs with x~0.3

CsPb(Br/Cl)_3_ NCs with size ~30 nm were prepared by partial anion-exchange of CsPbBr_3_ NCs^57,58^.

First, ∼30 nm-sized CsPbBr_3_ NCs were synthesized with the following modifications with respect to Ref. ^59^. The injection of Cs-oleate and further annealing were conducted at 225 °C. Such large CsPbBr_3_ NCs were colloidally stabilized by lecithin as a capping ligand. While the reaction mixture was cooling down, at 50 °C 2 ml of lecithin in toluene (0.01 M) were added and the solution was stirred for 30 min. The crude solution was centrifuged for 5 min at 1600 $\times$ g. The resulting supernatant was discarded and the precipitate was dispersed in a lecithin/toluene mixture (0,01 M, 2 mL) and kept undisturbed for 1 h. Then, the solution was centrifuged a second time for 4 min at 1600 $\times$ g. The precipitate was redispersed in toluene/1,2-dichlorobenzene (v/v 0.6:1).

The anion-exchange reaction was conducted at room temperature. As a source of chloride ions the mixture of didodecyldimethylammonium chloride (DDAC)/PbCl_2_ in toluene was used. The solution of DDAC/PbCl_2_ in toluene was prepared by mixing 42 mg DDAC and 13.8 mg PbCl_2_ with 7.5 mL toluene under moderate heating. 200 µL of CsPbBr_3_ NC colloid prepared as described above were mixed with 6.5 µL of the DDAC/PbCl_2_ mixture.

An estimation x ~ 0.3 for the relative amount of chloride atoms in CsPb(Cl_x_Br_1-x_)_3_ NCs is derived from the empirical Vegard’s law^60,61^, using the room temperature bandgap of this alloy$\,{E}_{{{{{{\rm{gap}}}}}}}\left({{{{{\rm{x}}}}}}\right)$ = 2.57 eV measured from the absorption spectrum.

A TEM image of these NCs is presented in Supplementary Fig. 13.

## Supplementary information

Supplementary Information

Peer Review File

## Data Availability

All data that support the conclusions of this study are included in the article and the Supplementary Information file. These data are available from the corresponding author upon request.

## Code availability

Numerical codes used in this study are available from the corresponding author upon request.
