# Author Correction: Universal scaling laws for charge-carrier interactions with quantum confinement in lead-halide perovskites

**DOI:** 10.1038/s41467-023-36513-0

**Published:** 2023-02-09

**Authors:** Philippe Tamarat, Elise Prin, Yuliia Berezovska, Anastasiia Moskalenko, Thi Phuc Tan Nguyen, Chenghui Xia, Lei Hou, Jean-Baptiste Trebbia, Marios Zacharias, Laurent Pedesseau, Claudine Katan, Maryna I. Bodnarchuk, Maksym V. Kovalenko, Jacky Even, Brahim Lounis

**Affiliations:** 1 Université de Bordeaux, LP2N, Talence, F-33405 France; 2 Institut d’Optique and CNRS, LP2N, Talence, F-33405 France; 3 Empa-Swiss Federal Laboratories for Materials Science and Technology, CH-8600 Dübendorf, Switzerland; 4 Institute of Inorganic Chemistry, Department of Chemistry and Applied Biosciences, ETH Zürich, CH-8093 Zürich, Switzerland; 5 Univ Rennes, ENSCR, CNRS, ISCR-UMR 6226, Rennes, F-35000 France; 6 Univ Rennes, INSA Rennes, CNRS, Institut FOTON—UMR 6082, F-35000 Rennes, France

**Keywords:** Nanoparticles, Electronic properties and materials, Quantum dots

Correction to: *Nature Communications* 10.1038/s41467-023-35842-4, published online 16 January 2023

In this article the author name Yuliia Berezovska was incorrectly written as Yullia Berezovska. The original article has been corrected.

