# Supplementary material for: Universal scaling laws for charge-carrier interactions with quantum confinement in lead-halide perovskites
Source: Nat Commun. 2023 Jan 16;14:229. doi: 10.1038/s41467-023-35842-4 (PMC9842747; doi:10.1038/s41467-023-35842-4)
Supplement: Supplementary file 1 — Supplementary Information [file 41467_2023_35842_MOESM1_ESM.pdf]

Supplementary Information

Universal scaling laws for charge-carrier interactions with quantum confinement in lead-halide perovskites

Tamarat and al.

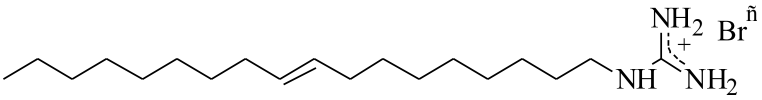

Supplementary Fig. 1. Structural formula of N-(octadec-9-en-1-yl)guanidinium hydrobromide (oleylguanidinium bromide, OGB) ligands.

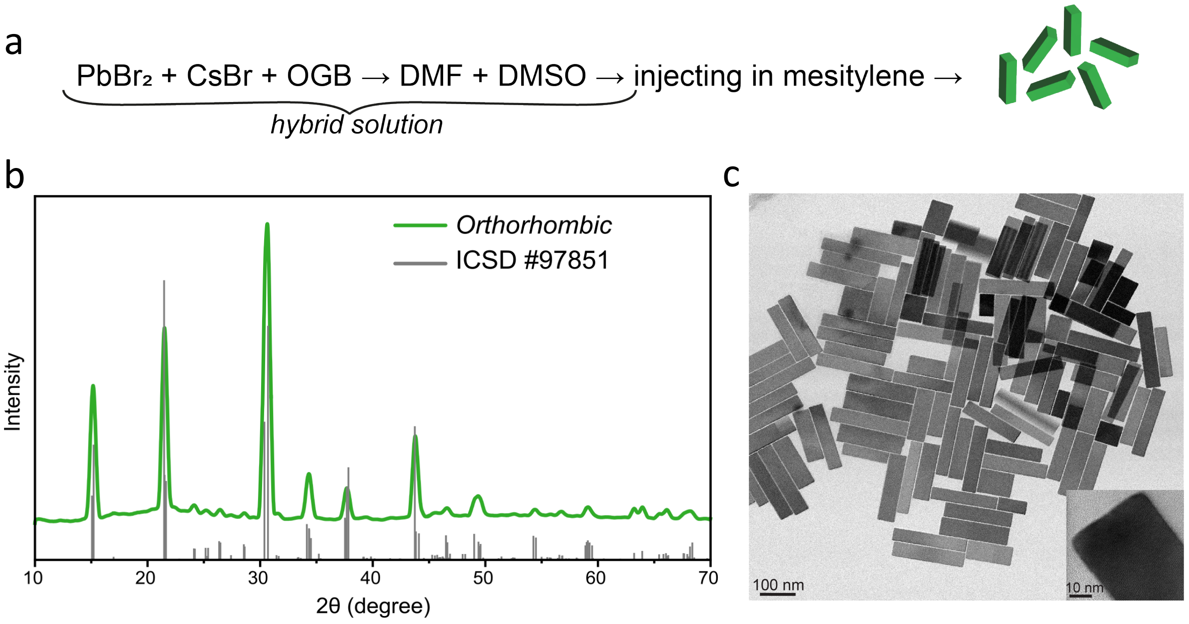

Supplementary Fig. 2: Synthesis and characterization of the OGB-capped $CsPbBr_3$ NCs. **a**, Schematic illustration of the synthetic procedure. **b**, Powder X-ray diffraction pattern of $CsPbBr_3$ NCs purified by ethyl acetate points to an orthorhombic crystal phase. **c**, Transmission electron microscopy images of the OGB-capped $CsPbBr_3$ NCs.

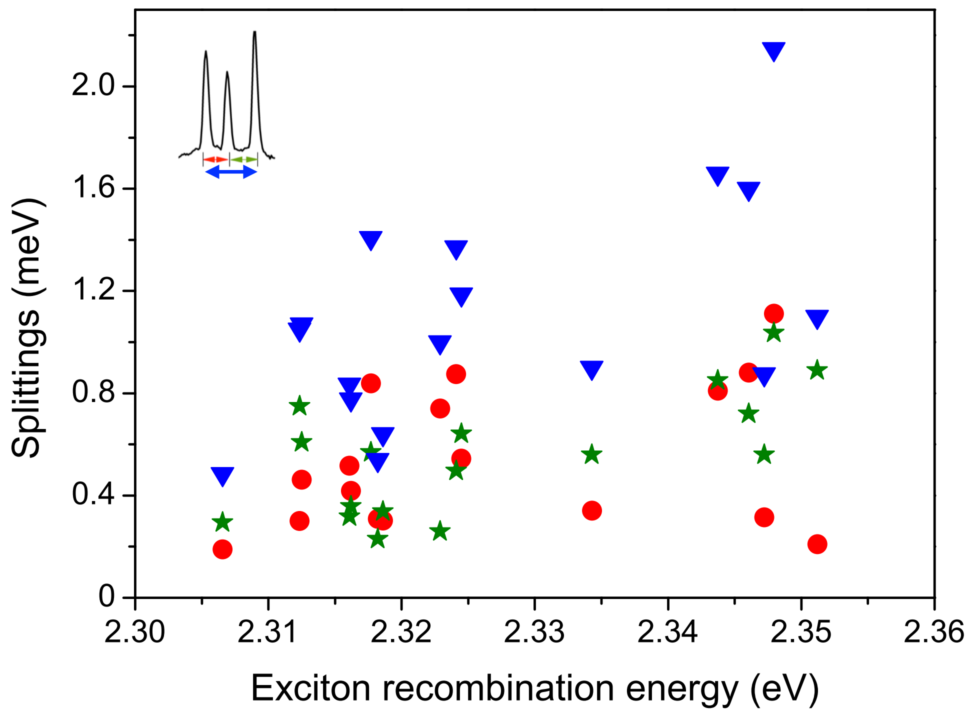

Supplementary Fig. 3: Statistics of bright triplet splittings of nearly-bulk $CsPbBr_3$ NCs. The red disks (resp. green stars) are the splittings between the two lowest (resp. highest) energy ZPLs, while the blue triangles are the splittings between the extreme triplet ZPLs. These splittings are plotted as a function of the exciton recombination energy, taken at the central ZPL.

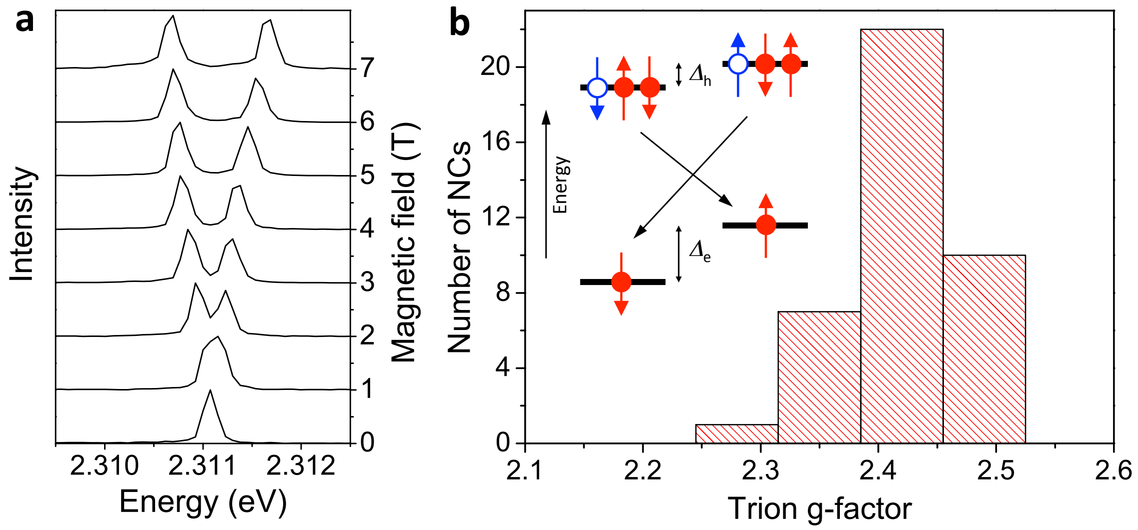

Supplementary Fig. 4: Trion Zeeman splitting and distribution of trion g-factors.
a, Representative magnetic field dependence of the PL spectrum of a $CsPbBr_3$ NC displaying trion emission. **b**, Histogram of the trion g-factors of 41 single NCs. Inset: Scheme of energy levels and recombination transitions for a negative trion under magnetic fields. The red (blue) arrows with solid (hollow) circles represent the spin states of electrons (holes). The level splitting associated with the hole and the electron are noted Δ_h and Δ_e, respectively.

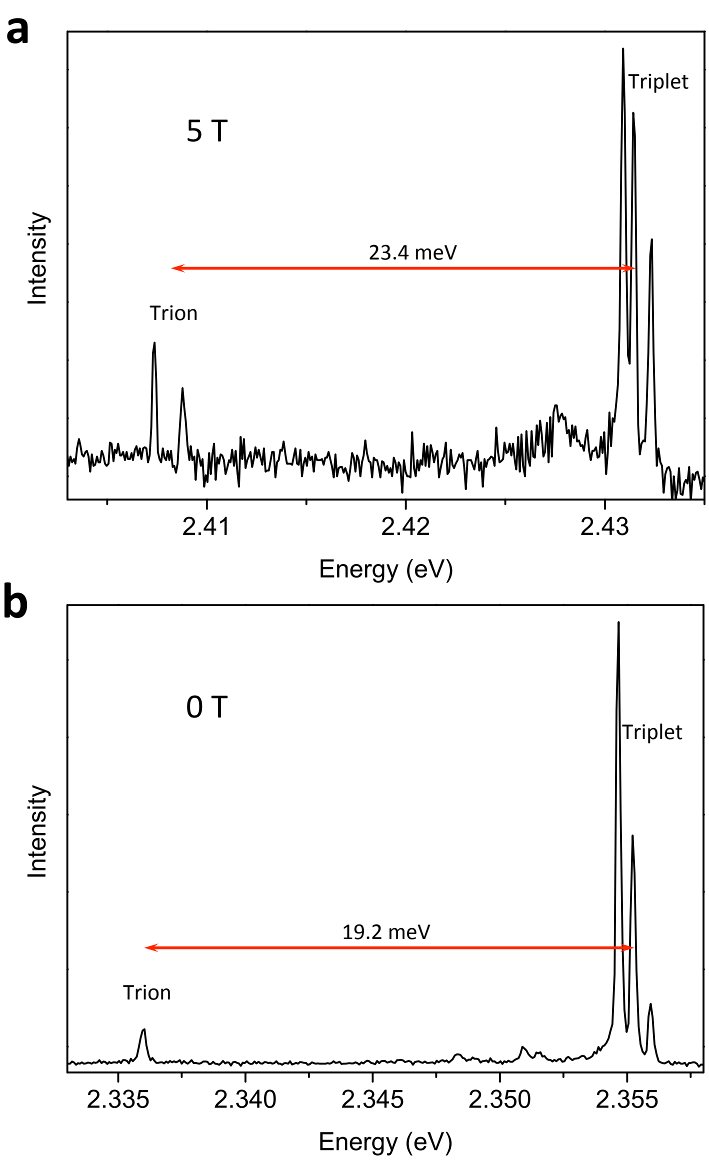

Supplementary Fig. 5: Trion binding energy in 5.5 nm sized and 14.5 nm sized $CsPbBr_3$ NCs.

a, PL spectrum of a 5.5 nm sized single $CsPbBr_3$ NC at 3.5 K and under a magnetic field of 5 T. The offset between the trion Zeeman doublet and the exciton central triplet ZPL is 23.4 meV. **b**, PL spectrum of a 14.5 nm sized single $CsPbBr_3$ NC at 3.0 K in zero field. The offset between the trion line and the exciton central triplet ZPL is 19.2 meV.

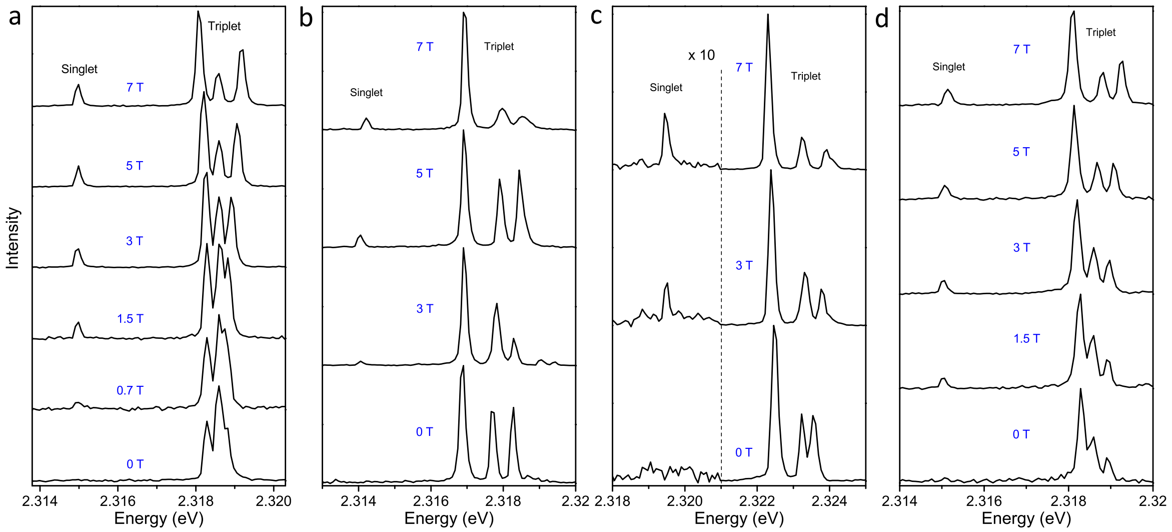

Supplementary Fig. 6: Magnetic brightening of the ground dark singlet state of single $CsPbBr_3$ NCs.

a, **b**, **c**, **d**, Evolution of the PL spectrum of four different single NCs with magnetic field. The emergence of a red-shifted ZPL under magnetic fields is the hallmark of a field-induced mixing between an optically forbidden state and a neighboring bright state. This red-shifted ZPL is attributed to the ground dark singlet exciton state of $CsPbBr_3$ NCs. The spectra displayed in (**a**) have been recorded on the same NC as the one presented in Fig. 2a.

The relative weights of the ZPLs not only depend on the corresponding radiative lifetimes and state populations, but also on the collection efficiency of the associated dipole radiation patterns. As a consequence, the weight of the singlet ZPL under magnetic brightening results from many factors: The population of the singlet state, which is set by the nonradiative relaxation from the high-energy photo-excited states; The bright-dark magnetic coupling, which depends on the orientation of the NC with respect to the magnetic field; The collection efficiency for the corresponding transition-dipole radiation pattern, which depends on the NC orientation with respect to the optical axis. These factors explain the large distribution of singlet-ZPL weights throughout our study.

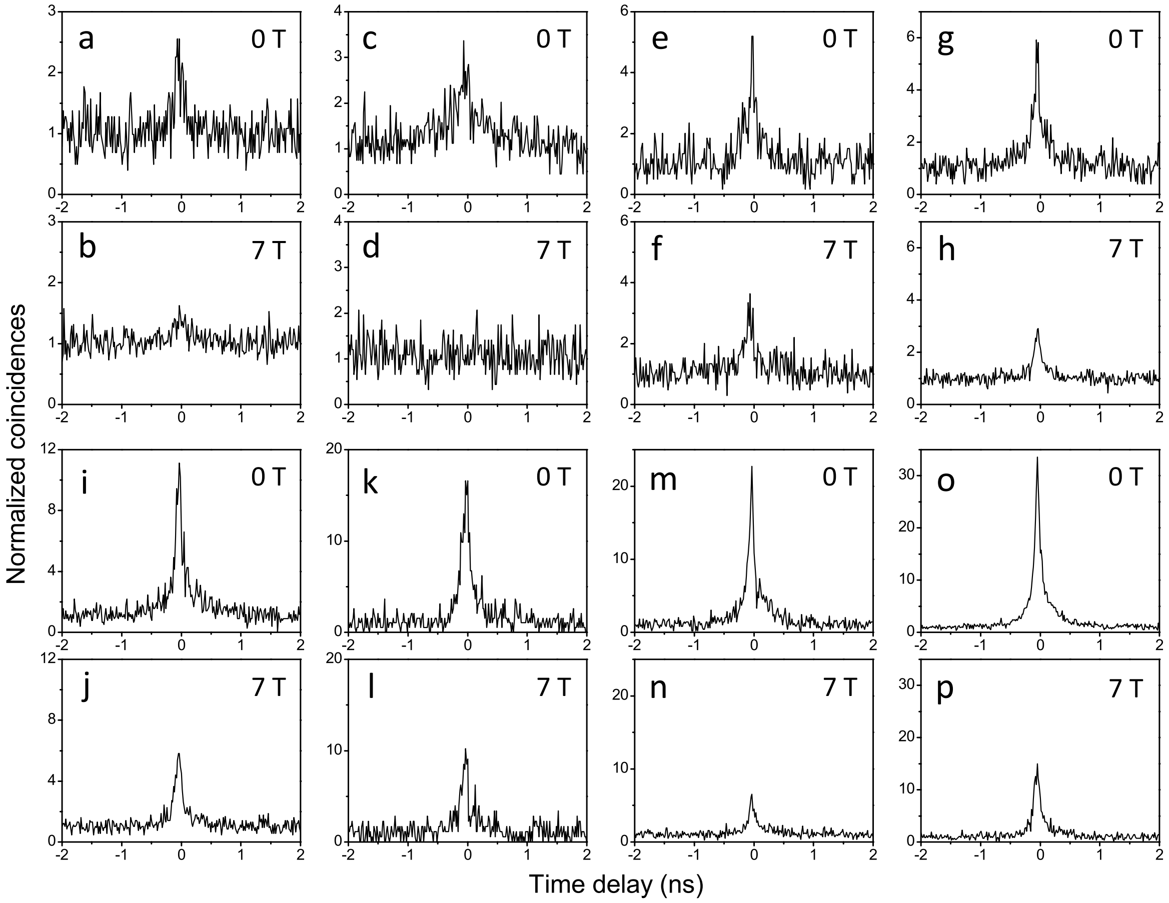

Supplementary Fig. 7: Photon correlation histograms of single nearly-bulk $CsPbBr_3$ NCs.

The PL intensity autocorrelation functions of 8 single NCs measured at 3.5 K and in zero field are assorted in ascending order of zero-delay autocorrelation $g^{(2)}(0)$ in **a**, **c**, **e**, **g**, **i**, **k**, **m**, **o**. The values of $g^{(2)}(0)$ range from 2.5 up to 32. Their corresponding autocorrelation functions at 7 T are presented in **b**, **d**, **f**, **h**, **j**, **l**, **n**, **p** with the same vertical scales, showing evidence for a reduction of photon bunching effect under magnetic field. These behaviors have been observed for all nearly-bulk $CsPbBr_3$ NCs. Note that the slight asymmetry of the autocorrelation histograms m and o may be due to a residual cross-talk effect between the two avalanche photodiodes.

As explained and modeled in Ref. [1], photon bunching takes its origin in the fact that the long-lived ground state stores the exciton for its long lifetime, which promotes the generation of biexcitons in the NC, followed by biexciton to zero-exciton two-photon cascades. The weakening of photon bunching with the application of magnetic fields results from the shortening of the dark state lifetime.

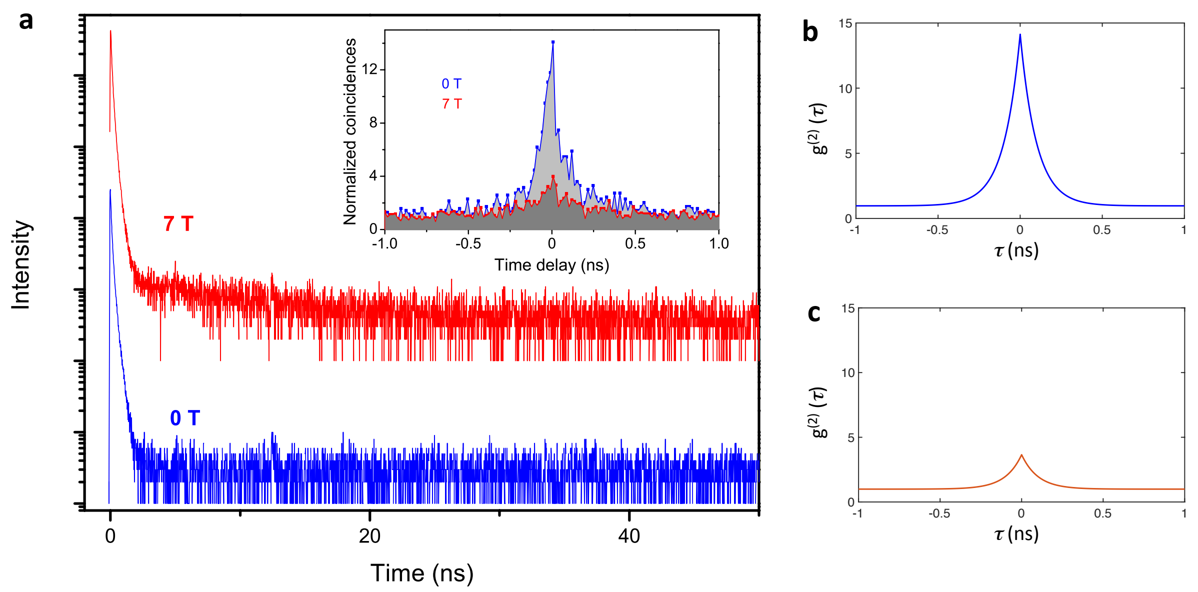

Supplementary Fig. 8: PL decay and photon coincidence histograms under magnetic fields.

a, Correlated PL decays and photon coincidence histograms for the same single NC at 0 T and at 7 T, the temperature being 3.5 K. This NC is the one whose PL spectra are displayed in Supplementary Fig. 6b. The correlations are well reproduced in **b**, **c** with the model developed in Ref. [1], taking $\Gamma_B = 10$ ns^{-1}, $\gamma_0 = 0.1$ ns^{-1}, $W = 0.025$ ns^{-1}, $\eta_{XX} = 0.12$, $\Gamma_D = 0.01$ ns^{-1} at 0 T (**b**) and $\Gamma_D = 0.125$ ns^{-1} at 7 T (**c**) (i.e. a lifetime of 8 ns given by the long component of the PL decay in **a**).

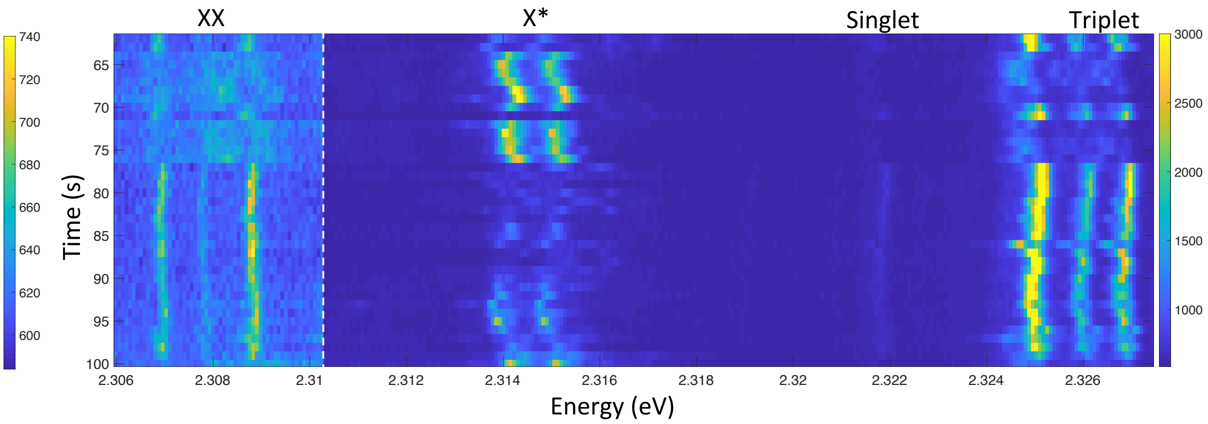

Supplementary Fig. 9: Spectral trail of a NC at 7 T under high excitation intensity

These series of consecutive PL spectra were recorded at 50 kW cm^{-2} on the same NC as in Fig. 3a. One can notice that the trion binding energies experience a much larger spread than the biexciton ones in Fig. 4. Since the NCs are either in the neutral or in the charged state, the exciton and trion spectral features are inherently measured on different emission spectra. Spectral jumps occurring during these emission switches are frequent and may lead to dispersion in the measured trion binding energies. Charge complexes such as trions are indeed highly sensitive to the local dielectric environment and to charge redistributions located at the surface of the NC.

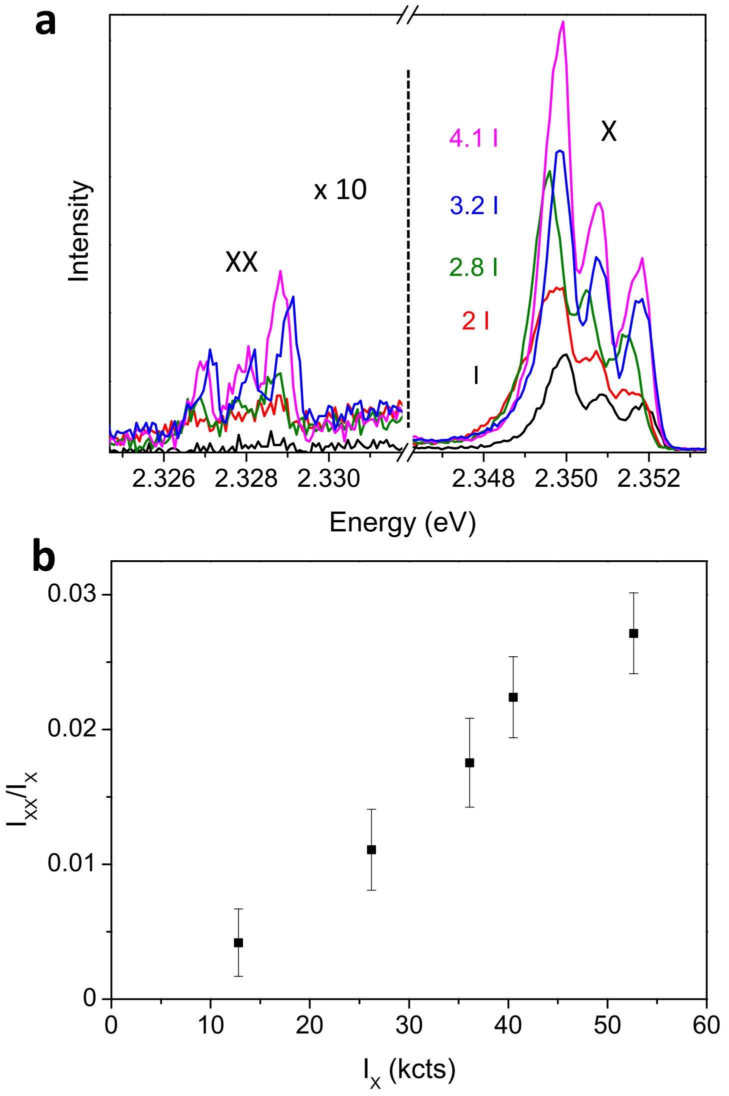

Supplementary Fig. 10: Dependence of the PL spectra on the excitation intensity.
a, Evolution of the spectral features assigned to the exciton triplet (X) and the biexciton-to-exciton triplet (XX) under increasing excitation intensities ranging from I = 0.8 kW cm^{-2} to 4.1 I. **b**, The ratio of the integrated intensities of the XX and X spectral structures is plotted as a function of the integrated intensities of the X triplet and shows a linear behavior, as a distinctive signature of XX biexciton emission.

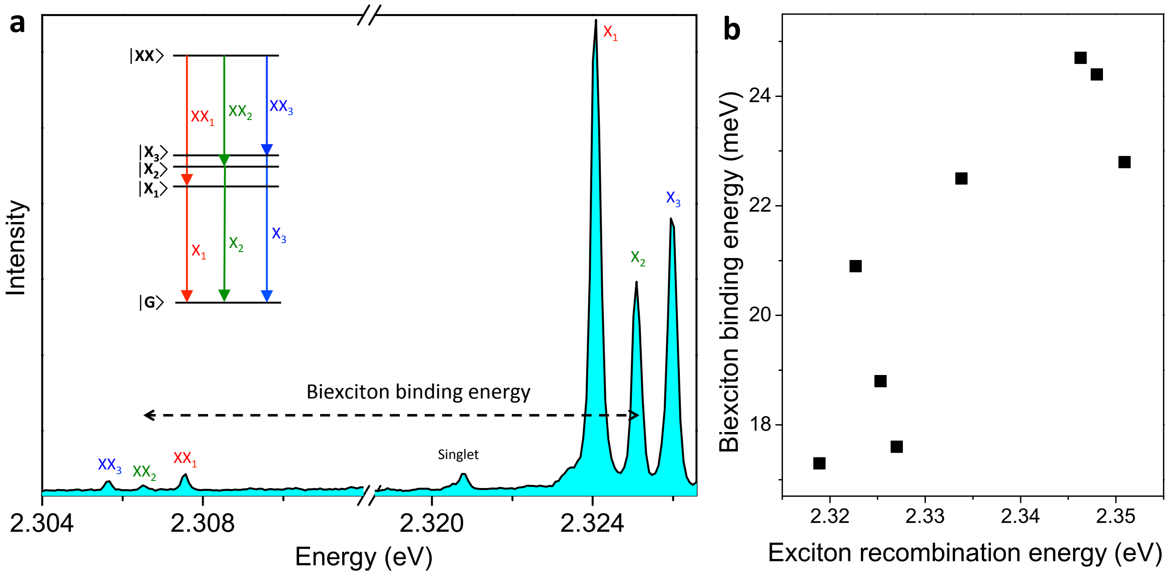

Supplementary Fig. 11: Distribution of the biexciton binding energy in $CsPbBr_3$ large NCs.

a, Low-temperature PL spectrum of a single NC at high excitation intensity (50 kW cm^{-2}) and 7 T. The four lines at energies higher than 2.32 eV are assigned to recombination ZPLs of the triplet (named X_1, X_2, X_3) and the dark ground singlet. The red-shifted lines named XX_1, XX_2, XX_3 are assigned to the biexciton-to-exciton transitions, as shown in the inset. **b**, Distribution of the biexciton binding energy, measured as the energy offset of the XX_2 line with respect to the X_2 line, as a function of the exciton recombination energy (taken at the X_2 line).

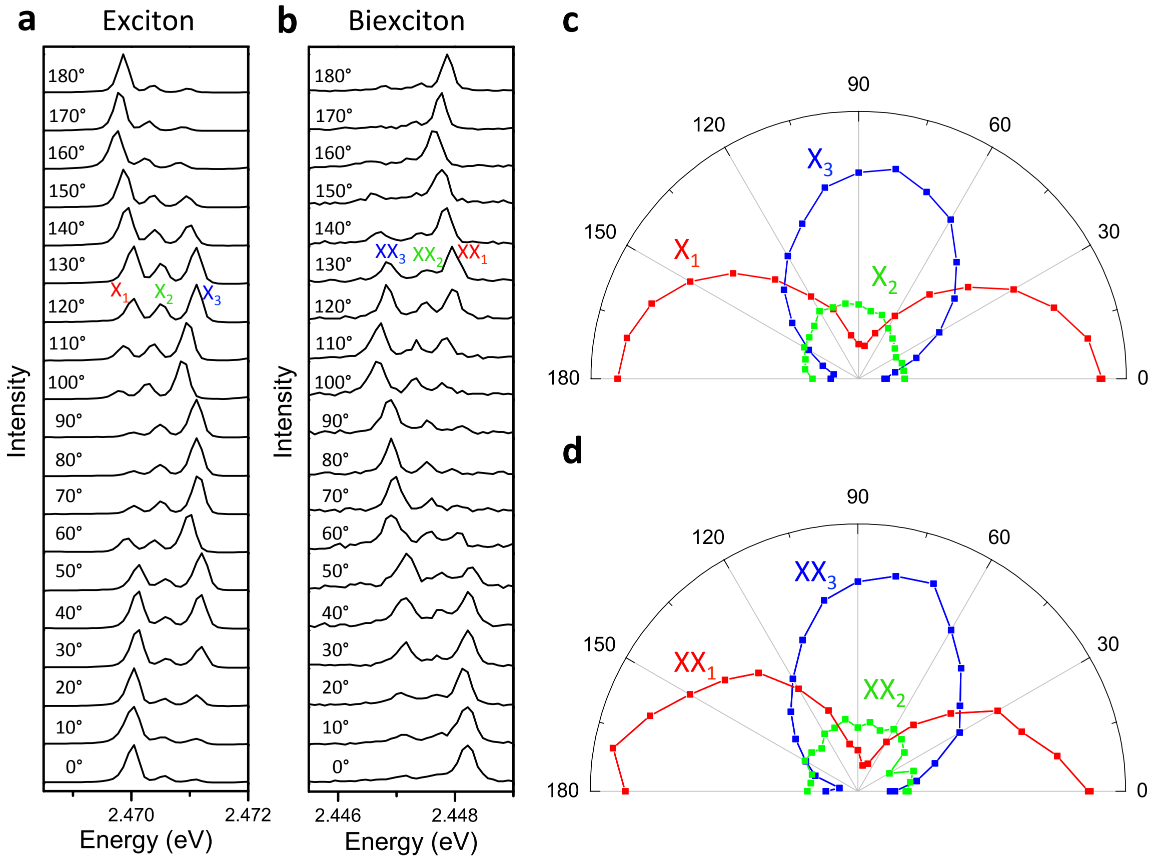

Supplementary Fig. 12: Polarization of the exciton and biexciton recombination lines. **a**,**b** Polarized PL spectra of a single $CsPb(Cl_xBr_{1-x})_3$ single NC with x~0.3 and size ~30 nm (See Methods and Supplementary Fig. 13), for various analyzer angles ranging from 0° to 180°. The series of spectra in **a** are zoomed on the exciton line triplet (X_1, X_2, X_3 ZPLs), while those in **b** are zoomed on the biexciton recombination ZPLs (named XX_1, XX_2, XX_3). The evolutions of the exciton and biexciton ZPL-intensities as a function of the polarizer angle are displayed in the polar plots **c** and **d**, respectively. The polar plot in **c** shows evidence that the transition dipoles associated to X_1 and X_3 ZPLs have nearly linear and orthogonal polarizations in the focal plane, while the weak X_2 line with a weak polarization character is compatible with a transition dipole oriented along the optical axis. The striking similarity between the polar plots provides further evidence of the correspondence between the biexciton-to-exciton and exciton-to-zero-exciton transitions, as a result of symmetry (See Supplementary Note 2).

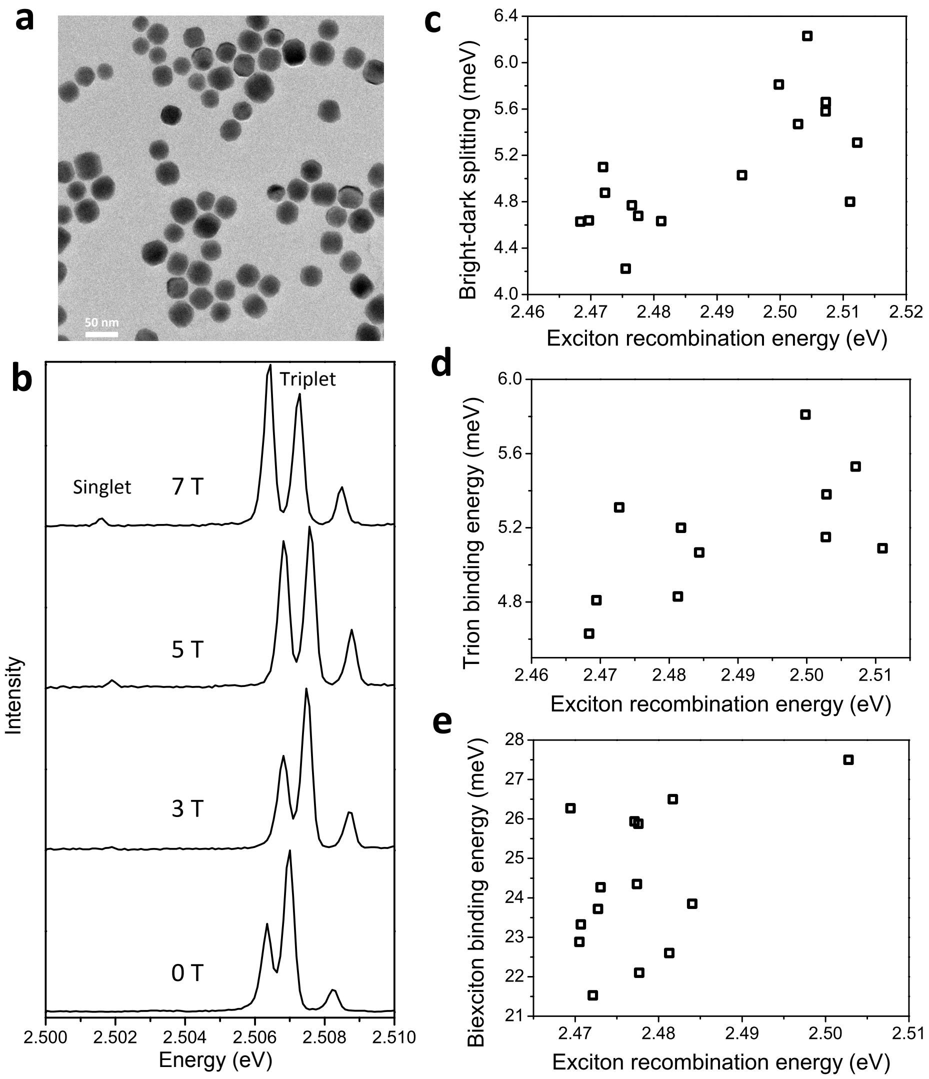

Supplementary Fig. 13: magneto-optical spectroscopic data on single $CsPb(Cl_xBr_{1-x})_3$ NCs with x~0.3.

a, TEM image of these NCs, which have an average NC size ~30 nm. **b**, Example of low-temperature PL spectrum of a single NC, showing evidence for magnetic brightening of the ground dark singlet state. **c**, The exciton bright-dark splittings are found in the range 5-6 meV. **d**, Distribution of trion binding energies, measured from the red-shift of the trion line with respect to the central exciton triplet line. **e**, Distribution of biexciton binding energies, measured from the red-shift of the biexciton triplet with respect to the central exciton triplet line. An example of biexciton spectral feature in these NCs is shown in Supplementary Fig. 12.

Material	Band gap E_{gap} (eV)	Binding energy R_{y} (meV)	Eff. Bohr radius a_{B} (nm)	Reduced mass μ (m_{e})	Eff. Dielectric constant ε
$FAPbBr_3$ (2K)	2.233[2]	24[2]	3.87	0.115[2]	8.4[2]
$CsPbI_3$	1.723[3]	15 ± 1[3]	4.64	0.114[3]	10.0[3]
$CsPbBr_3$	2.342[3]	33 ± 1[3]	3.07	0.126[3]	7.3[3]
$CsPb(Cl_{0.24}Br_{0.76})_3$	2.513	42	2.51	0.148	7.1
$CsPbCl_3$	3.056[4]	64 ± 1.5[4]	1.72	0.202[4]	6.6[4]

Supplementary Table 1: Fundamental parameters used for the normalization of the charge-complex binding energies and the dark-bright exciton splitting.

This table indicates the electronic band gap E_{gap} (eV), the bulk exciton binding energy R_{y} (meV), the effective Bohr radius a_{B} (nm), the reduced mass μ given in electron mass (m_{e}) units, and the effective dielectric constant ε. Except for $CsPb(Cl_{0.3}Br_{0.7})_3$ with mixed halides, the values of E_{gap} and R_{y} are taken from low-temperature magneto-optical measurements [2-4]. The effective mass and the effective dielectric constant were derived from the hydrogen model combined with either (i) the approximation $\mu = (m_{\mathrm{e}} E_{\mathrm{gap}})/4P^2$, using estimations of the Kane momentum P [2,3] or (ii) the dependence of the exciton diamagnetic-shift coefficient on μ and $\varepsilon_{\mathrm{eff}}$ [4]. For the mixed halide compound $CsPb(Cl_xBr_{1-x})_3$ with x~0.24, the low-temperature band gap is estimated using a linear Vegard's interpolation law $E_{\mathrm{gap}}(\mathrm{x}) = \mathrm{x}\, E_{\mathrm{gap}}(\mathrm{CsPbCl_3}) + (1-\mathrm{x}) E_{\mathrm{gap}}(\mathrm{CsPbBr_3})$. The values of R_{y} and a_{B} are obtained from the empirical relations[4] $R_{\mathrm{y}} \propto E_{\mathrm{gap}}{}^{1.62}$ and $a_{\mathrm{B}} \propto E_{\mathrm{gap}}{}^{-1.33}$. Similarly, $\mu \propto E_{\mathrm{gap}}$ and $\varepsilon \propto (R_{\mathrm{y}} a_{\mathrm{B}})^{-1} \propto E_{\mathrm{gap}}{}^{-0.29}$ provide estimates for the reduced mass and effective dielectric constant.

Supplementary Note 1

1. Smearing of the electronic and vibrational density of states in bulk perovskites

We address the possible origins for the variation of emission energies among different single $CsPbBr_3$ NCs, especially down to the bulk limit (Fig. 2). Recent experimental investigations on bulk 3D perovskites combining various techniques looking at several orders of magnitude for the dynamics (Neutron, Raman and Brillouin scattering, NMR, dielectric and ultrasonic measurements) have indeed shown that besides soft acoustic and polar damped vibrations, ultraslow structural relaxation exists in these bulk materials. Its microscopic origins may be stochastic deviations of cation positions or torsions of the halogen octahedra, which translate into a complex potential landscape felt by the exciton. This is a consequence of the very strong lattice anharmonicity or intrinsic polymorphous nature of the perovskite structure[5-7]. A first conventional theoretical approach consists in performing ab-initio molecular dynamics (AIMD). Quarti et al. [8] reported early in 2015 strong stochastic and localized band gap fluctuations over time for 3D bulk halide perovskites using AIMD. It can be further refined in the constant-temperature, constant-pressure ensemble (NPT) to capture the coupling of disorder to acoustic-like fluctuations[9]. These band gap fluctuations are reminiscent of the spectral diffusion of the emission. However, AIMD's calculations have strong limitations. The computational cost is especially prohibitive, with various limitations on the total computational time and hence the quality of the structural dynamics sampling, the size of the simulation super-cell, the lattice temperature (high temperature are very often used to artificially speed up the simulations) and the level of theory used, especially to extract accurate information on the physics of the electron-phonon coupling.

In order to get a broader view on the general consequences of these fluctuations, a new theoretical framework is proposed to extract the smearing of the vibrational and electronic density of states, which leads to a broadening of the distribution of the emission energies. In the Supplementary Fig. 14, we present the effect of structural disorder on the phonons and electronic structure of $CsPbBr_3$. Supplementary Fig. 14a compares the phonon spectral function (color map) and phonon dispersion (black lines) calculated using the polymorphous (ground state structural disorder) and ideal monomorphous (nuclei fixed at their high-symmetry positions) cubic networks, respectively. The polymorphous description was shown recently to give a much better description of XRD pair distribution functions. Interestingly, accounting for static disorder in $CsPbBr_3$ leads to a large smearing of the optical phonons, displaying an over-damped anharmonic behavior across the reciprocal lattice similar to the one observed experimentally by various phonon spectroscopy techniques. Supplementary Fig. 14b compares the associated electron spectral function (color map) and band structure (black curves) calculated using the polymorphous and monomorphous networks, respectively, including the effect of spin-orbit coupling. For the calculation of the electron spectral function, we have included the effect of electron-phonon coupling at 0 K using the special displacement method[10,11]. Note that the electronic band-gap between black curves in underestimated at the theoretical level of DFT. Nevertheless, apart from a remarkable band-gap increase arising from both structural disorder and electron-phonon coupling, our computations reveal a considerable smearing of the electron states, which allows various optical transitions. To get a more quantitative agreement for the effective band gap, this new methodology could be improved by relying on other functionals, but the main physical conclusion concerning the smearing of both vibrational and electronic densities of states will not be changed.

It is well established that in hybrid perovskite crystals, the various orientations of the electric dipole of the organic molecular cation play an important role in creating structural electrostatic potential fluctuation. Such fluctuations are also present in inorganic perovskites (without polar molecules) as a result of stochastic deviations of cation positions or torsions of

the halogen octahedra (Ref.[12] and references therein). The wavefunction of the exciton center-of-mass evolves in the resulting potential landscape and may be spatially localized in its minima at low temperatures. Such potential wells confining the exciton should have a typical size larger than the exciton Bohr radius and thus be in little number in the large $CsPbBr_3$ NCs of this study. This picture is supported by the experimental observation of exciton diffusion towards low energy localized states in 2D perovskites[13,14]. Furthermore, exciton localization towards the lowest-energy potential wells was found to occur at low temperature[14].

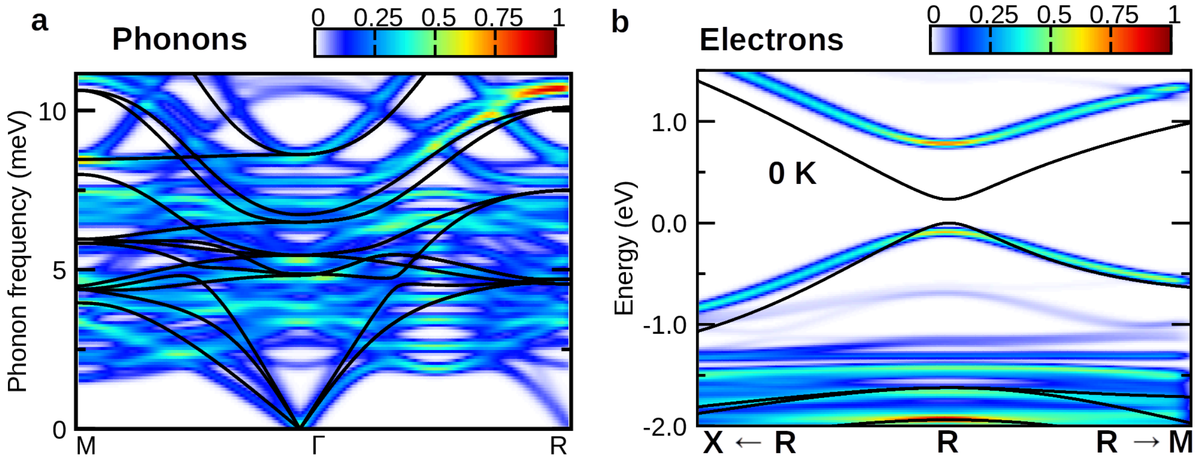

Supplementary Fig. 14: Calculated phonon and electron spectral functions.
a, Phonon spectral function of cubic polymorphous (disordered) $CsPbBr_3$ (color map) for frequencies up to 11 meV, plotted along the M-Γ-R high-symmetry path of the reciprocal lattice space. **b**, Electron spectral function at 0 K of cubic polymorphous $CsPbBr_3$ (color map) plotted along the X-R-M high-symmetry path of the reciprocal lattice space. Black lines represent the phonon dispersion (**a**) and electron band structure (**b**), calculated using the monomorphous cubic 'ideal' and undistorted network, i.e. without accounting for structural disorder. Ab-initio calculations were performed within the density functional theory (DFT), using plane waves as basis functions in the PBEsol approximation[15], as implemented in the electronic structure package Quantum Espresso[16]. We also used optimized norm-conserving Vanderbilt pseudopotentials as proposed by Haman[17]. We started our calculations from the monomorphous structure, using the unit cell of cubic $CsPbBr_3$ (5 atoms) with the nuclei fixed at their high symmetry positions (space group $Pm\bar{3}m$). The lattice constant was fixed to the DFT-PBEsol relaxed value of 5.874 Å and the plane wave energy cutoff was set to 120 R_y. To obtain the polymorphous structures[6] of cubic $CsPbBr_3$ we relax the system to its disordered ground state in a 2×2×2 supercell. To this aim, we initially displaced the nuclei away from their high-symmetry positions using special displacements[10,11] generated for T = 0 K, and allow the system to relax until the residual force component per atom was less than 3×10^{-4} eV/ Å. The sampling of the Brillouin zone of the unit cell and 2×2×2 supercell was performed using uniform 6×6×6 and 3×3×3 **k**-grids, respectively. The phonons of the monomorphous and polymorphous structures were obtained by means of the frozen-phonon method[18] and Fourier interpolation of the dynamical matrices. To account for electron-phonon coupling effects on the electronic structure of the polymorphous network, we used the special displacement method[10,11], enforcing a smooth Berry connection between the phonon eigenmodes as implemented in the ZG branch of the EPW code[19]. Phonon and electron spectral functions of the polymorphous structure, shown as color maps, were evaluated using the band structure unfolding technique as described in Ref.[10].

2. Exciton fine structure and exciton dark-bright splitting

In the case of the dark-bright splitting of the exciton, a specific dimensional analysis is needed. As shown in previous studies conducted on $FAPbBr_3$ [20] and $CsPbI_3$ [1] perovskites, the dark-bright splitting Δ_{BD} is essentially set by the long-range component of the electron-hole exchange interaction, which is directly related to the dimensionless ratio $L_{\mathrm{eff}}/a_{\mathrm{B}}$ and to the difference between the energies of longitudinal and transverse excitonic polaritons $\hbar\omega_{\mathrm{LT}}/3$, where

$$\hbar\omega_{\mathrm{LT}} = \frac{2}{3\pi\varepsilon a_{\mathrm{B}}^3}\left(\frac{e\hbar p_{\mathrm{cv}}}{m_0 E_{\mathrm{gap}}}\right)^2 \qquad (1) \ ,$$

p_{cv} being the Kane interband momentum matrix element and m_0 the electron mass[21]. Indeed, since the seminal paper of Onodera and Toyozawa[22], it is recognized that the problems of exchange interaction and transverse and longitudinal excitons are connected[23]. Thus, $\hbar\omega_{\mathrm{LT}}$ shall afford a more adequate normalization factor of the dark-bright splitting as compared to the exciton Rydberg energy R_{y}. This quantity can be related to R_{y} and E_{gap} considering the Kane energy $E_{\mathrm{Kane}} = p_{\mathrm{cv}}{}^2/(2m_0)$ introduced as a fundamental constant of 2D [24] and 3D [25] perovskites. It was experimentally shown that a single Kane energy is enough to describe the evolution of the effective masses with bandgaps for various compositions of 3D bulk metal halide perovskites[2,3]. Using $E_{\mathrm{Kane}}/E_{\mathrm{gap}} \propto \mu^{-1}$ leads to

$$\hbar\omega_{LT} \ \propto \frac{E_{\mathrm{Kane}}}{E_{\mathrm{gap}}{}^2\varepsilon\, a_{\mathrm{B}}^3} \propto \frac{1}{\mu E_{\mathrm{gap}}\varepsilon a_{\mathrm{B}}^3} \propto \frac{R_{\mathrm{y}}{}^2}{E_{\mathrm{gap}}} \qquad (2) \ ,$$

the proportionality coefficients being material independent. Therefore, using the dimensionless quantity $\Delta_{\mathrm{BD}}/\left(R_{\mathrm{y}}{}^2/E_{\mathrm{gap}}\right)$ should lead to a universal dependence of the exciton dark-bright splitting as a function of the quantum confinement. This is demonstrated in Fig. 4c, where a clear correlation between the dimensionless bright-dark splitting and the exciton recombination energy, after bandgap subtraction, is evidenced.

We now justify why a scaling factor designed to account for the long-range exchange interaction remains relevant for an experimental quantity related to the sum of the short-range and long-range contributions. We have numerically inspected the bulk limits of both contributions for various perovskites. Rather than considering a few values spread in the literature[26] for the short-range component, we use instead the general bulk formula proposed by Ben Aich et al.[27] and derived from Rössler et al. [28]. $\Delta_{SR,BD} = \frac{2}{3}D = \frac{2}{3}\frac{C}{\pi a_B^3}$. Indeed, this formula is proposed in the same spirit as the present work and aims at proposing a law based on an empirical factor C=107.6 meV.nm^3. This contribution is computed in the Supplementary Table 2, together with the bulk limit of the long range part $\Delta_{LR,BD,bulk} = \frac{\hbar\omega_{LT}}{3}$, for all compounds relevant to perovskite nanostructures. Interestingly, when the proper scaling factor $R_{\mathrm{y}}{}^2/E_{\mathrm{gap}}$ is introduced, all these perovskites yield very similar dimensionless bulk limits of the exchange energy in the 5-6 range, in reasonable agreement with the bulk-limit extrapolation of the experimental master curve (Fig. 4c). Since the short-range and long-range contributions of the exchange interaction are predicted to have the same NC size-dependence in the strongly confined regime[21], the applicability of the scaling factor $R_{\mathrm{y}}{}^2/E_{\mathrm{gap}}$ over the whole range of confinement regimes is strengthened.

	$CsPbI_3$	$MAPbI_3$	$FAPbI_3$	$CsPbBr_3$	$MAPbBr_3$	$FAPbBr_3$	$CsPb(Br,Cl)_3$ (x_{Cl}=0.24)
$\Delta_{SR,BD}$ (meV)	0.22917	0.20950	0.13795	0.79542	0.58725	0.39425	1.44394
$\Delta_{SR,BD}/\left({R_{\mathrm{y}}}^{2}/E_{\mathrm{gap}}\right)$	1.6359	1.34511	1.05199	1.79446	1.67507	1.80153	2.05704
$\Delta_{LR,BD,bulk}$ (meV)	0.61302	0.64849	0.52006	1.57754	1.18363	0.75214	2.48142
$\Delta_{LR,BD,bulk}/\left({R_{\mathrm{y}}}^{2}/E_{\mathrm{gap}}\right)$	4.37597	4.16367	3.96570	3.55891	3.37618	3.43692	3.53503
$\Delta_{BD,bulk} = \Delta_{SR,BD} + \Delta_{LR,BD,bulk}$ (meV)	0.84220	0.85799	0.65801	2.37296	1.77088	1.14639	3.92536
$\Delta_{BD,bulk}/\left({R_{\mathrm{y}}}^{2}/E_{\mathrm{gap}}\right)$	6.01190	5.50878	5.01769	5.35337	5.05125	5.23845	5.59207

Supplementary Table 2: Contributions of the short-range and long-range electron-hole exchange interactions, using the normalization factor ${R_{\mathrm{y}}}^{2}/E_{\mathrm{gap}}$.

Supplementary Note 2

Correlated polarizations for the biexciton and exciton emissions

This section aims at providing the explanations for the correlation between polarizations of the biexciton-to-exciton and exciton-to-ground-state transition lines (Supplementary Fig. 12). As already discussed in Ref.[29], the double group irreducible representations of the D_{2h} point group are suitable for the orthorhombic bulk phase of $CsPbBr_3$ and lead to the following decomposition of the exciton fine structure:

$$E_{1/2,\mathrm{u}} \otimes E^*_{1/2,\mathrm{g}} = A_\mathrm{u} \oplus B_{1\mathrm{u}} \oplus B_{2\mathrm{u}} \oplus B_{3\mathrm{u}} \quad (3)$$

For biexciton states related to the same bulk Bloch states, one obtains:

$$E_{1/2,\mathrm{u}} \otimes E^*_{1/2,\mathrm{g}} \otimes E_{1/2,\mathrm{u}} \otimes E^*_{1/2,\mathrm{g}} = 4\left(A_\mathrm{g} \oplus B_{1\mathrm{g}} \oplus B_{2\mathrm{g}} \oplus B_{3\mathrm{g}}\right) \quad (4)$$

The A_g irreducible representation is the only one related to a total momentum equal to zero corresponding to a complete filling of the doubly degenerated conduction band edge, and thus to the biexciton ground state.
A transition from the A_g biexciton ground state to one of the three bright exciton states as described by $\langle \varphi_\mathrm{biexc} | H_{D.E.} | \varphi_\mathrm{exc} \rangle$ corresponds to an optical transition with a polarization along one of the three (x, y, z) crystallographic axes, since the vectorial representation V relevant for the dipolar electric Hamiltonian $H_\mathrm{D.E.}$ is $V = B_{1\mathrm{u}}(z) \oplus B_{2\mathrm{u}}(y) \oplus B_{3\mathrm{u}}(x)$. This transition is therefore followed by an optical transition from an exciton state to the ground state $\langle \varphi_\mathrm{exc} | H_\mathrm{D.E.} | \varphi_\mathrm{G} \rangle$ with the same polarization, because the ground state also behaves as the totally symmetric representation A_g.

Supplementary References

1. Tamarat, P. *et al.* The dark exciton ground state promotes photon- pair emission in individual perovskite nanocrystals. *Nature Communications* **11,** 1–8 (2020).
2. Galkowski, K. *et al.* Determination of the exciton binding energy and effective masses for methylammonium and formamidinium lead tri-halide perovskite semiconductors. *Energy & Environmental Science* **9,** 962–970 (2016).
3. Yang, Z. *et al.* Impact of the Halide Cage on the Electronic Properties of Fully Inorganic Cesium Lead Halide Perovskites. *ACS Energy Lett.* **2,** 1621–1627 (2017).
4. Baranowski, M. *et al.* Exciton binding energy and effective mass of $CsPbCl_3$: a magneto-optical study. *Photonics Research* **8,** A50–A55 (2020).
5. Weadock, N. J. *et al.* Test of the Dynamic-Domain and Critical Scattering Hypotheses in Cubic Methylammonium Lead Triiodide. *Physical Review Letters* **125,** 075701 (2020).
6. Zhao, X.-G., Dalpian, G. M., Wang, Z. & Zunger, A. Polymorphous nature of cubic halide perovskites. *Physical Review B* **101,** 155137 (2020).
7. Hehlen, B. *et al.* Pseudospin-phonon pretransitional dynamics in lead halide hybrid perovskites. *Physical Review B* **105,** 024306 (2022).
8. Quarti, C., Mosconi, E. & De Angelis, F. Structural and electronic properties of organo-halide hybrid perovskites from *ab initio* molecular dynamics. *Physical Chemistry Chemical Physics* **17,** 9394–9409 (2015).
9. Carignano, M. A. *et al.* A close examination of the structure and dynamics of $HC(NH_2)_2PbI_3$ by MD simulations and group theory. *Phys. Chem. Chem. Phys.* **18,** 27109 (2016).
10. Zacharias, M. & Giustino, F. One-shot calculation of temperature-dependent optical spectra and phonon-induced band-gap renormalization. *Physical Review B* **94,** 075125 (2016).
11. Zacharias, M. & Giustino, F. Theory of the special displacement method for electronic structure calculations at finite temperature. *Physical Review Research* **2,** 013357 (2020).
12. Yaffe, O. *et al.* Local Polar Fluctuations in Lead Halide Perovskite Crystals. *Physical Review Letters* **118,** 136001 (2017).
13. Kinigstein, E. D. *et al.* Edge States Drive Exciton Dissociation in Ruddlesden- Popper Lead Halide Perovskite Thin Films. *ACS Materials Letters* **2,** 1360–1367 (2020).
14. Baldwin, A. *et al.* Local Energy Landscape Drives Long-Range Exciton Diffusion in Two-Dimensional Halide Perovskite Semiconductors. *J. Phys. Chem. Lett.* **12,** 4003–4011 (2021).
15. Perdew, J. P. *et al.* Restoring the Density-Gradient Expansion for Exchange in Solids and Surfaces. *Physical Review Letters* **100,** 136406 (2008).
16. Giannozzi, P. *et al.* Advanced capabilities for materials modelling with Quantum ESPRESSO. *J. Phys.: Condens. Matter* **29,** 465901 (2017).
17. Hamann, D. R. Optimized norm-conserving Vanderbilt pseudopotentials. *Physical Review B* **88,** 085117 (2013).
18. Kunc, K. & Martin, R. M. *Ab initio calculation of phonon spectra*. (Plenum, New York, 1983).
19. Poncé, S., Margine, E. R., Verdi, C. & Giustino, F. EPW: Electron–phonon coupling, transport and superconducting properties using maximally localized Wannier functions. *Computer Physics Communications* **209,** 116–133 (2016).
20. Tamarat, P. *et al.* The ground exciton state of formamidinium lead bromide perovskite nanocrystals is a singlet dark state. *Nat Mater* **18,** 717–724 (2019).
21. Goupalov, S. V. & Ivchenko, L. E. Electron-hole long-range exchange interaction in

semiconductor quantum dots. *Journal of Crystal Growth* **184/185,** 393–397 (1998).
22. Onodera, Y. & Toyozawa, Y. Excitons in Alkali Halides. *J. Phys. Soc. Jpn.* **22,** 833–844 (1967).
23. Denisov, M. M. & Makarov, V. P. Longitudinal and Transverse Excitons in Semiconductors. *Phys. stat. sol. (b)* **56,** 9–59 (1973).
24. Even, J., Pedesseau, L., Dupertuis, M. A., Jancu, J. M. & Katan, C. Electronic model for self-assembled hybrid organic/perovskite semiconductors: Reverse band edge electronic states ordering and spin-orbit coupling. *Physical Review B* **86,** 205301 (2012).
25. Even, J., Pedesseau, L. & Katan, C. Analysis of Multivalley and Multibandgap Absorption and Enhancement of Free Carriers Related to Exciton Screening in Hybrid Perovskites. *J. Phys. Chem. C* **118,** 11566–11572 (2014).
26. Becker, M. A. *et al.* Bright triplet excitons in caesium lead halide perovskites. *Nature* **553,** 189–193 (2018).
27. Ben Aich, R., Ben Radhia S, Boujdaria, K., Chamarro, M. & Testelin, C. Multiband k·p Model for Tetragonal Crystals: Application to Hybrid Halide Perovskite Nanocrystals. *J. Phys. Chem. Lett.* **11,** 808–817 (2020).
28. Rössler, U. & Trebin, H.-R. Exchange and polaron corrections for excitons in the degenerate-band case. *Physical Review B* **23,** 1961–1970 (1981).
29. Fu, M. *et al.* Neutral and Charged Exciton Fine Structure in Single Lead Halide Perovskite Nanocrystals Revealed by Magneto-Optical Spectroscopy. *Nano Letters* **17,** 2895–2901 (2017).
